# Fatal community-acquired *Bacillus cereus* pneumonia in an immunocompetent adult man: a case report

**DOI:** 10.1186/s12879-019-3836-3

**Published:** 2019-02-27

**Authors:** Ryosuke Ishida, Kazunori Ueda, Tadashi Kitano, Tomohiko Yamamoto, Yasuyoshi Mizutani, Yutaka Tsutsumi, Koji Imoto, Yuji Yamamori

**Affiliations:** 1 Department of Emergency and Critical Care Medicine, Shimane Prefectural Central Hospital, 4-1-1 Himebara, Izumo, Shimane 693-8555 Japan; 2 Department of Pathology, Shimane Prefectural Central Hospital, Izumo, Japan; 3 Department of Cardiology, Shimane Prefectural Central Hospital, Izumo, Japan; 4 Department of Molecular Oncology, Fujita Health University School of Medicine, Toyoake, Aichi Japan; 5 Department of Pathology, Haruhi Respiratory Medical Hospital, Kiyosu, Aichi Japan

**Keywords:** *Bacillus cereus*, Community-acquired infection, Anthrax-like toxin

## Abstract

**Background:**

*Bacillus cereus* is a gram-positive rod bacterium that is responsible for food poisoning. It is naturally widely distributed, and thus often contaminates cultures. Although it is rarely considered responsible, it can cause serious infections under certain conditions. However, lethal infections, especially in immunocompetent patients, are rare.

**Case presentation:**

A healthy 60-year-old man developed community-acquired *B. cereus* pneumonia and alveolar hemorrhage unveiled by abrupt chest pain and hemoptysis with no other advance symptoms. *B. cereus* induced silent alveolar destruction without any local or systemic inflammatory response. Although the lesion resembled lung anthrax, there was no evidence of *Bacillus anthracis* toxin.

**Conclusions:**

Some isolates of *B. cereus* can cause anthrax-like fulminant necrotizing pneumonia in immunocompetent patients. If this type of *B. cereus* were used as a means of bioterrorism, it may be quite difficult to recognize as bioterrorism. We should keep *B. cereus* in mind as a potential pathogen of fulminant human infectious disease.

## Background

*Bacillus cereus* is a ubiquitous, gram-positive rod bacterium that is responsible for food poisoning in humans [1, 2]. *B. cereus* is naturally widely distributed, and thus often contaminates cultures. Although it is rarely responsible for serious infections, previous reports have demonstrated that it can cause serious infections under certain conditions [1, 2]. However, lethal infections, especially in immunocompetent patients, are rare. Recently, it has been shown that some *B. cereus* contain the plasmid coding *Bacillus anthracis* toxin genes, which induces toxin-mediated severe necrotizing pneumonia [2, 3]. We report a case of fatal community-acquired *B. cereus* pneumonia and alveolar hemorrhage in a healthy man, unveiled by abrupt chest pain and hemoptysis with no other advance symptoms. Here, *B. cereus* induced silent alveolar destruction without any local or systemic inflammatory response. Since pathological findings showed anthrax-like lung lesion, we tried to determine whether this *B. cereus* strain contained *B. anthracis* toxin genes using real-time polymerase chain reaction (PCR).

## Case presentation

A 60-year-old man presented with sudden severe right shoulder and flank pain and numbness of the right hand. The patient had a history of working in his home garden every day. He had no subjective symptoms prior to the day of admission, and no past medical history other than hypertension, which was managed with medication. The patient called an ambulance 3 h after the onset of symptoms and was able to get into the ambulance unassisted. He was transported to a nearby hospital. At the hospital, he developed hemoptysis and hypoxemia with severe forced breathing and tachypnea. He was tracheally intubated and transferred to our emergency department by air ambulance helicopter 6 h after the onset of symptoms.

On examination in our emergency department, a coarse crackle with right lateral dominance was audible. A small volume of blood was continuously suctioned through the tracheal tube, although bronchoscopic examination did not reveal any source of bleeding. The patient’s blood pressure was 132/87 mmHg, pulse was 109 beats per minute and body temperature was 36.7 °C. He was mechanically ventilated with spontaneous breathing at a rate of 14 breaths per minute under sedation. No skin eruptions or lesions were observed.

**Fig. 1:**
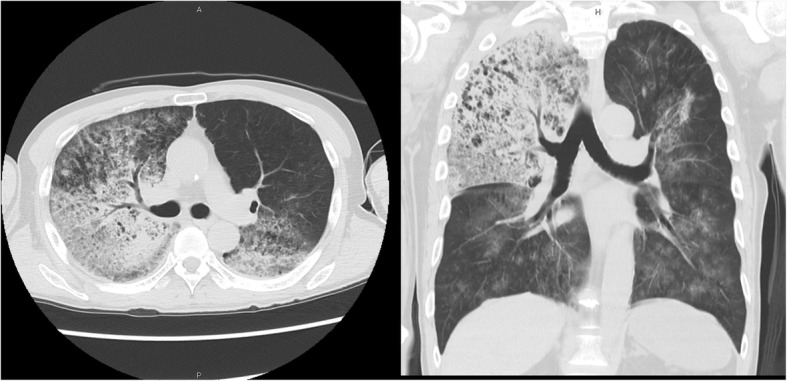
Chest CT showing infiltration predominantly in the right upper lobe and spreading to the right middle and lower lobe and left hilar area, suggesting alveolar hemorrhage

Upon examination of chest computed tomography (CT), we saw infiltration predominant in the right upper lobe and spreading to the right middle and lower lobe and left hilar area (Fig. 1). Peripheral blood was collected for laboratory examination. Arterial blood gas analysis showed a pH of 7.174, with a partial pressure of carbon dioxide of 62.4 mmHg, a partial pressure of oxygen of 94.3 mmHg, a base deficit of − 7.4. under the condition of end-expiratory pressure at 10 cm H_2_O, and a fraction of inspired oxygen of 0.5, indicating acute respiratory failure. Other laboratory data were normal, including blood cell count, coagulation, and biochemistry, including inflammatory biomarkers, other than a slight elevation in serum creatinine level (1.37 mg/dL).

Electrocardiography showed a sinus rate of 86 beats per minute, with an obvious ST segment elevation in the inferior leads. Echocardiography also showed severe hypokinesis of the cardiac inferior wall. The patient’s serum troponin T level was elevated (0.487 ng/mL).

The patient’s history was obtained from his family, and showed only hypertension. His current medications included enalapril, carvedilol, and amlodipine. He had no known allergies and no recent travel history. He did not smoke and there was no history of unusual ingestions. The Triage DOA® intoxication screening test result was negative.

From the laboratory results and other tests, there were two contradictory clinical concerns: revascularization of the coronary artery and alveolar hemostasis. As the etiology of the alveolar hemorrhage was unknown, we were obliged to seek the pathogenesis under mechanical ventilation, with no obvious indicators for a hemostatic approach. Thus, after discussion, we decided to prioritize the revascularization of the coronary artery. After heparinization, coronary angiography confirmed 99% severe stenosis with a flow delay (thrombolysis in myocardial infarction grade 2 flow) of the mid right coronary artery at segment 2. Thrombus aspiration was performed, followed by implantation of a drug-eluting stent (DES). To minimize the bleeding risk, we delayed administration of antiplatelet drugs, aspirin and prasugrel, until the time of definite decision to implant the DES.

Next, transcatheter arterial embolization was performed to treat the alveolar hemorrhage. Although we did not detect overt extravasation by angiography, we believed that the location of the hemorrhage was a branch of the right bronchial artery, which we embolized using a gelatin sponge. However, we were unable to control the alveolar hemorrhage, which increased and blew out from the tracheal tube, making it very difficult to maintain oxygenation and circulation. The patient died 12 h after the onset of symptoms. No antibiotics were administered during treatment.

Autopsy was performed with the family’s consent immediately after the patient’s death.

The following day, additional laboratory blood exams revealed that negative for the anti-neutrophil cytoplasmic antibody, anti-nuclear antibody, and anti-glomerular basement membrane antibody. Levels of lung surfactant proteins A and D, as well as KL-6, were normal. Later, *B. cereus* was cultured from the sputum sample suctioned through the tracheal tube.

Immunohistochemistry of *B. cereus* and real-time PCR for pXO1-like plasmid from lung tissue were performed to confirm that the bacterium was *B. cereus* and whether this bacterium produced anthrax-like toxin.

The lungs were fixed in 20% formalin for 24 h and embedded in paraffin, followed by pathological examination. *B. cereus* immunostaining was performed using anti-*Bacillus cereus* rabbit polyclonal antibody (Abcam, Cambridge, UK).

Next, we performed DNA extraction and real-time PCR for *B. anthracis* toxin plasmid. Two pieces of 10 μm-thick Formalin fixed paraffin embedded (FFPE) sections were collected in Eppendorf tubes. DNA was extracted from these sections with the use of Nucleospin DNA FFPE XS kit (Macherey-Nagel, Düren, Germany), according to the manufacturer’s instruction. For detecting infection with *B. cereus* containing pXO1-like plasmid, lethal factor (LF) gene (Genbank M29081.1) and protective antigen gene (PAg) (Genbank AF268967.1) were amplified by real-time PCR. For amplifying LF, two primer sets were prepared.: LF1, 5′- CAGCTTTATGCACCGGAAGC-3′ (forward) and 5′- CGCTCCAGTGTTGATAGTGC-3′ (reverse), generating a product of 148 bp; and LF2, 5′- TCAGCTTAAGGAACATCCCACA -3′ (forward) and 5′- GCTTCCGGTGCATAAAGCTG-3′ (reverse), generating a product of 144 bp. PAg was amplified using the primers 5′- CAGGCTCGAACTGGAGTGAA -3′ (forward) and 5′- TCACTAGGATTAACCGCCGC -3′ (reverse), generating a product of 118 bp. PCR reactions were carried out in a 25-μL final volume containing 2 μL of sample DNA, 12.5 μl of 2× reaction mixture (QuantiTect SYBR Green PCR Kits; Qiagen, Hilden, Germany) and 0.2 μM primers. The real-time PCR was performed with Rotor Gene Q (Qiagen), with an initial holding step at 95 °C for 15 min, followed by 50 cycles of three-step PCR (94 °C for 15 s, 55 °C for 30 s, and 72 °C for 30 s) with SYBR Green fluorescence monitoring to detect amplification. The melting curve was examined to check for contamination. As a positive control, genomic DNA of *Bacillus anthracis* (JNBP01251) was provided by the Gifu Type Culture Collection, Graduate School of Medicine, Gifu University.

**Fig. 2:**
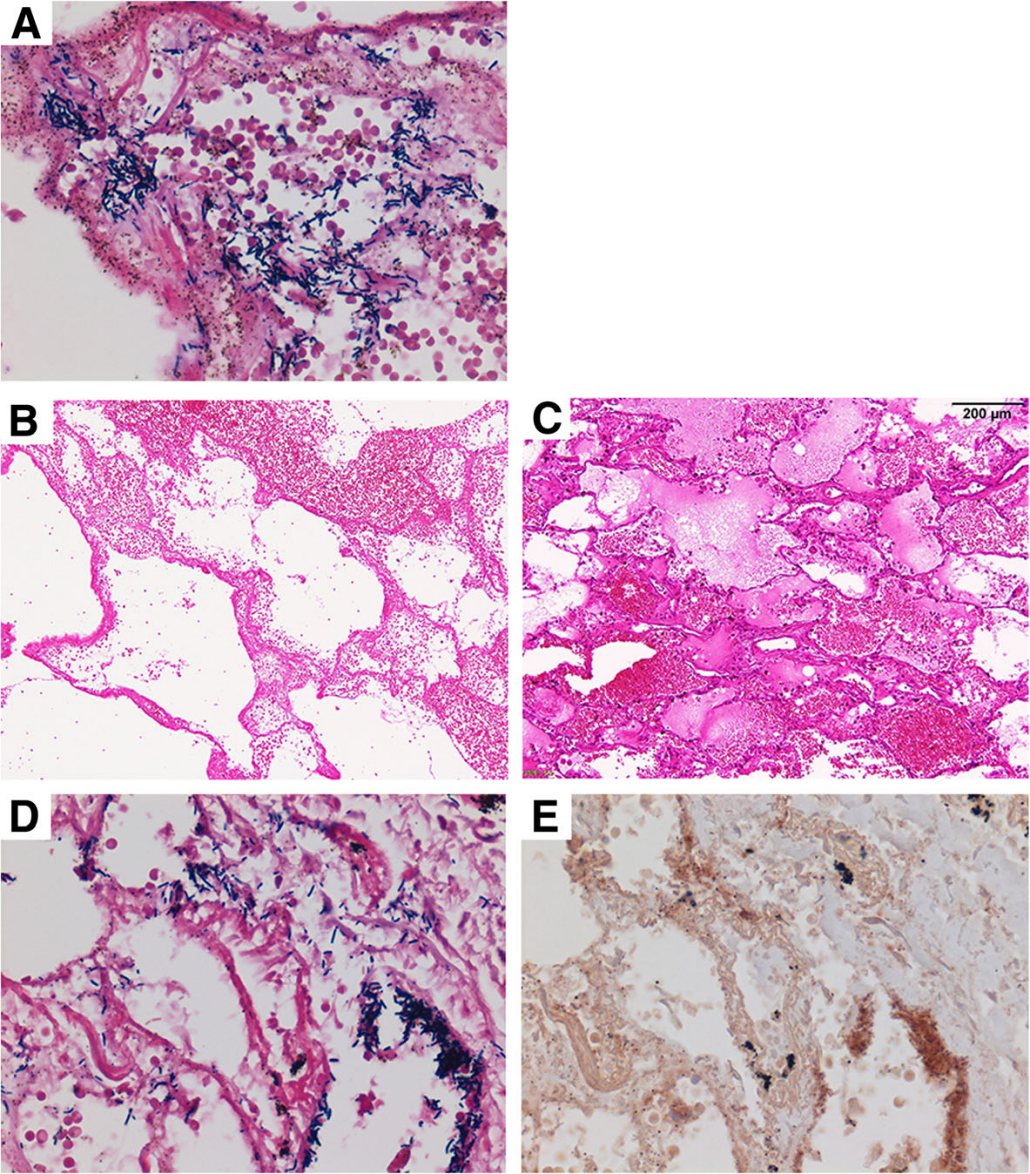
Histologic sections of the lung taken at autopsy. Tremendous proliferation of gram-positive rods on the alveolar wall. Severe necrosis of the alveolar wall and capillary vessel without proliferation of neutrophils (A). Hemorrhagic diffuse alveolar damage within the hyaline membrane, and pulmonary edema (B, C). The dotted immunoreactivity of the *B. cereus* antibody was observed on the bacterium and spores (D, E)

Histologic sections of the lung, especially of the right upper lobe, demonstrated necrotizing hemorrhagic pneumonia similar to anthrax, with tremendous proliferation of gram-positive rods. The bacteria were diffusely gram-positive. Additionally, hemorrhagic diffuse alveolar damage within the hyaline membrane that was probably due to acute respiratory distress syndrome was also observed throughout the lungs. The bacteria reacted to the *B. cereus* antibody, and did not react to *Pseudomonas aeruginosa* and *Escherichia coli* antibodies. There was no infiltration of neutrophils. There was also no deposition of immunoglobulins or complements on the alveolar walls by immunofluorescence, excluding a diagnosis of vasculitis. *B. cereus* was also confirmed from the sputum culture. Therefore, *B. cereus* necrotizing pneumonia was confirmed pathologically (Fig. 2).

In the real-time PCR, amplification was obtained in the positive control (*B. anthracis* DNA), but not in the patient sample or the negative control (no template).

## Discussion and conclusion

*Bacillus cereus* infection generally causes food poisoning, although it can cause fulminant disease in an immunocompromised host. Miyata and colleagues summarized 16 *B. cereus* pneumonia cases and concluded that most of these cases occurred in patients with hematological disorders or alcohol abuse [4]. However, it has been previously shown that, even in immunocompetent patients, *B. cereus* may induce serious necrotizing infections [1]. For example, Sliman et al. [5] determined that localized infection by *B. cereus* in the eye or viscera, such as pneumonia, may precipitate severe necrotizing infection with profound morbidity. Generally, *B. cereus* and *Bacillus anthracis* genomes have a high homology [6, 7]. Recently, it has been shown that some isolates of *B. cereus* contain *B. anthracis* toxin genes, which are accountable for toxin-mediated severe necrotizing pneumonia [2, 3]. Hoffmaster et al. [8] demonstrated that *B. cereus* isolated from patients with life-threatening pneumonia had a circular plasmid called pBCXO1. This plasmid shows 99.6% similarity with the plasmid pXO1, which encodes a *B. anthracis* toxin. Furthermore, previous reports presented two healthy welders in Louisiana [9] and two healthy metalworkers in Texas [10] who died of *B. cereus* pneumonia, and the pXO1 plasmid was confirmed in the Texas cases.

In the present case, there was no local infiltration of neutrophils, suggesting the observed necrotizing pneumonia was toxin-mediated and less inflammatory. However, we could not demonstrate that this *B. cereus* produced anthrax toxin.

Our pathological findings are consistent with lung anthrax [11]. We speculate that some *B. cereus* may produce toxins different from those produced by *B. anthracis*, but cause corresponding symptoms. This might be responsible for fatal *B. cereus* pneumonia. Here, we present some previous cases of fatal *B. cereus* pneumonia (Table 1). Our case is unique in that it was less inflammatory, displayed few symptoms, and was rapidly progressive. In previous reports, the subjects presented with symptoms such as nausea, vomiting, fever, chill, or white blood cell count elevation, suggesting bacterial infection, and infection was diagnosed in other cases at a high incidence [8, 9].

**Table 1:** Clinical presentations of anthrax and *Bacillus cereus* pneumonia in metalworkers, compared with present report

	Present case	Texas, 2003 [10]		Louisiana,1997 [9]		Louisiana, 1994 (*n* = 1) [8]	Cases of inhalation anthrax in 2001 (*n* = 10) [17]
Patient characteristic		Patient 1	Patient 2	Patient 1	Patient 2		
Immunocompromised	No	No	No	No	No	No	No
Illicit drug use	No	No	No	No	No	No	No
Smoking	No	No	Yes	No	No	No	No
Other underlying complications	No	No	No	No	No	No	No
No. of days from symptom onset to medical care	1	6–7	8	5	3	2	3.5 (1–7)
Fever/chills	No	Yes	Yes	Yes	Yes	Yes	10
Dyspnea	Yes	Yes	Yes	Yes	Yes	Yes	8
Hemoptysis	Yes	Yes	Yes	Yes	Yes	Yes	0
Nausea/vomiting or diarrhea	No	Yes	Yes	No	No	Yes	9
Cough	Yes	Yes	Yes	Yes	Yes	Yes	9
Initial chest radiograph with effusions or infiltrates	Yes	Yes	Yes	Yes	Yes	Yes	10
Initial chest radiograph with widened mediastinum	No	No	No	No	No	No	7
Initial WBC count, cells/mm3	8400	15,100	25,100	26,900	8800	12,000	9800 (7500–13,300)
Hematocrit, %	44.7	61.9	57.4	Not Available	55.9	Not Available	46.0 (42.5–51.4)

*Note.* Adapted from Table 1 of Avashia SB, Riggins WS, Lindley C, et al. Fatal pneumonia among metalworkers due to inhalation exposure to *Bacillus cereus* containing *Bacillus anthracis* toxin genes. Clin Infect Dis 2007;44:414–6. WBC: white blood cell

*Data are median (range)

Unfortunately, we were unable to save our patient. However, we believe that there are factors in this case that may benefit clinicians in future cases, allowing them to save the patient’s life. At the initial presentation, physical examination and laboratory data indicated severe hypoxemia due to alveolar hemorrhage of unknown etiology and lack of inflammatory response. The patient had no previous indications of illness, had a regular routine, and the symptoms developed suddenly. Therefore, we did not consider infectious disease as an etiology. Rather, we considered a systemic disease, such as drug-induced alveolar hemorrhage or vasculitis.

In retrospect, the best treatment would have been immediate isolation of the right upper lobe, followed by optimal antibiotics. Additionally, selective blocking of the right upper bronchus may have delayed the increased expansion of the blood to the other lobe. However, we did not have sufficient information to determine that such a procedure was indicated, i.e., we could not determine that the lesion was localized within the right upper lobe.

The comorbid ST-elevation myocardial infarction (STEMI) complicated the case, presenting conflicting symptoms that required immediate attention (revascularization of the coronary artery and alveolar hemostasis). Since visible bleeding was minor, we estimated that the alveolar hemorrhage was controllable, and thus first treated the coronary artery. After discussion, we concluded that we could not continue the treatment while ignoring the inferior STEMI induced by severe stenosis at mid right coronary artery, which has high mortality rate [12]. Thus, we concluded that anti-platelet therapy was imperative. Venovenous extracorporeal membrane oxygenation could not be performed because of continuous massive bleeding.

The patient first presented with sudden severe right shoulder and flank pain. This may have been caused by acute myocardial infarction. However, his hemoptysis occurred simultaneously. Community-acquired pneumonia (CAP) has been previously reported to be associated with cardiovascular complications at a high incidence. Violi et al. reported that 32.2% of CAP patients experienced cardiovascular events after hospitalization, including 8% who experienced myocardial infarction. The risk increases as the Pneumonia Severity Index increases [13]. Although the mechanism is not totally elucidated, impaired endothelial function [14], activation of coagulation [15], and activation of platelets [16] are possible candidates. We speculate that the invasion of the *B. cereus* infection may have triggered the myocardial infarction. In conclusion, we report a case of severe necrotizing pneumonia caused by *B. cereus*. This is a rare case, as the patient was immunocompetent and asymptomatic until onset. Additionally, the environmental background of infection is totally different from those of previous reports. Some isolates of *B. cereus* can cause anthrax-like fulminant necrotizing pneumonia in immunocompetent patients. If this type of *B. cereus* were used as a means of bioterrorism, it may be quite difficult to recognize as bioterrorism. This is also a public health concern. Although we were not able to make a diagnosis in this case, we should keep *B. cereus* in mind as a potential pathogen of fulminant human infectious disease.

## Abbreviations

CAP: community-acquired pneumonia
CT: computed tomography
DES: drug-eluting stent
FFPE: Formalin fixed paraffin embedded
LF: lethal factor
PAg: protective antigen
PCR: polymerase chain reaction
STEMI: ST-elevation myocardial infarction
